# Explaining variability

**DOI:** 10.1590/2177-6709.21.6.015-016.edt

**Published:** 2016

**Authors:** David Normando

**Affiliations:** 1 Adjunct professor, Universidade Federal do Pará (UFPA), School of Dentistry, Belém, Pará, Brazil. Coordinator, Universidade Federal do Pará (UFPA), Graduate program in Dentistry, and ABO-Pará, Specialization course in Orthodontics, Belém, Pará, Brazil.

*"Is your guess as good as mine?"*

Lysle Johnston Jr.^1^, American orthodontist

Orthodontic treatment is routinely described to last, on average, 24 months. Nonetheless, every orthodontist has already experienced that this time length shows huge variability, depending on several factors that are inherent to the operator, the patient, scientific knowledge and mastering of the mechanics used in orthodontic movement. It is very likely that there is something else, something still unknown. In this case, as well as in many others, being aware of the mean time will end up being of minor importance and will produce meager information.^2^ Variability is routine in the biological field, a measure which is much more interesting than the mean itself. 

The vast majority of scientific investigations should be addressing the reasons of such variation in the results obtained, much more than they address the mean - a measure that encompasses the minority of individuals, most of the time. Finding out that a procedure reduces the mean treatment time by 10% is important, but it seems more interesting to explain why treatment variability among similar patients treated with the same procedure may exceed 100%.^3^ ^,^ ^4^

While statistical analyses aimed at comparing means are interesting, it would be much more fruitful modulating the factors associated to such variability. In that case, tests to compare means (*t* test, ANOVA) are left aside in order to let us use regression models that are capable of opening windows hardly glimpsed at. 

Let us provide a practical example. Researchers are carrying out a study to evaluate orthodontic treatment stability in Class III patients, mild or moderate, treated in a compensatory way at the permanent dentition. Any experienced orthodontist has already faced stable cases and relapse cases in this type of malocclusion. Suppose the authors observe that one out of four patients (25%) has had a clinical relapse of the malocclusion five years after the end of the treatment. That information is important, but is of less applicability in the clinical practice. If our next patient is given only that information, they would probably ask us: "- Do I have greater chances of stability or relapse?" 

A more individualized answer to that question would demand evaluating the several variables inherent to the patient, such as vertical facial pattern, preexisting degree of dentoalveolar compensation, the sagittal severity of the initial malocclusion, age at the beginning of the treatment, remaining growth potential and so on - since these are all factors that may have influence over the risk of treatment relapse. All these characteristics can be collected in a good diagnosis. Nevertheless, there are some others that should be added, with the purpose of enhancing the accuracy of the answer. Some can only be computed in the course of the treatment: the patient's cooperation taking care of the orthodontic appliances and the use of elastics, and regular appointment attendance; some others will be available only after the treatment, such as cooperation in using the retention and the residual growth. Even if we combine it all, there will still be something missing; however, the answer will be much more reliable than a cabalistic and unisonous number, 25%.

Statistical regression models allow us to model predictor variables of treatment stability and offer the next patient a more individualized analysis of relapse risks. By adding these variables to our analysis, be it clinical or statistical, we will have a more accurate answer to the patient's question. Instead of 25% of relapse risk for all of them, indistinctly, it will be possible to increase this risk rate for patients with certain characteristics, or reduce it for others.

Regression is, thus, an excellent tool for predicting what will happen during the treatment of your next patient or after its ending. A major part of what is named diagnosis and treatment plan in Orthodontics involves predicting. Surprisingly, many clinicians are aware of that, even though they are not fond of statistical models; whereas researchers should explore the benefits of multiple regression analyses more deeply, be in Orthodontics or other fields. 

David Normando - editor-in-chief (davidnormando@hotmail.com)
